# Effect of Treatment with Salsalate, Menhaden Oil, Combination of Salsalate and Menhaden Oil, or Resolvin D1 of C57Bl/6J Type 1 Diabetic Mouse on Neuropathic Endpoints

**DOI:** 10.1155/2016/5905891

**Published:** 2016-09-28

**Authors:** Matthew S. Yorek, Lawrence J. Coppey, Hanna Shevalye, Alexander Obrosov, Randy H. Kardon, Mark A. Yorek

**Affiliations:** ^1^Department of Veterans Affairs Iowa City VA Health Care System, Iowa City, IA 52246, USA; ^2^Veterans Affairs Center for the Prevention and Treatment of Visual Loss, Iowa City, IA 52246, USA; ^3^Department of Internal Medicine, University of Iowa, Iowa City, IA 52242, USA; ^4^Department of Ophthalmology and Visual Science, University of Iowa, Iowa City, IA 52242, USA; ^5^Fraternal Order of Eagles Diabetes Research Center, University of Iowa, Iowa City, IA 52242, USA

## Abstract

*Aims*. In this study a streptozotocin induced type 1 diabetes mouse model was used to assess the effectiveness of salsalate, menhaden oil, the combination of salsalate and menhaden oil, or resolvin D1 on neuropathic endpoints.* Materials and Methods*. Changes in body weight, blood glucose, serum markers for triglycerides, free fatty acids, cholesterol, and resolvin D1, motor and sensory nerve conduction velocities and thermal sensitivity were assessed, as well as performing in vivo confocal microscopy of subepithelial corneal nerves and immunohistochemistry of nerves in the cornea and foot pad.* Results*. Diabetic animals failed to gain weight and had elevated blood glucose levels. Diabetic mice had slowed nerve conduction velocity, reduced innervation of the foot pad and cornea subepithelial and epithelial layers, and reduced thermal sensitivity. Monotherapy treatment with salsalate, menhaden oil, and resolvin D1 reduced the pathological signs of diabetic neuropathy. The combination of salsalate and menhaden oil also reduced signs of pathology and generated elevated plasma levels of resolvin D1 compared to other groups.* Conclusions*. Additional studies are needed to determine whether the combination of salsalate and menhaden oil may be more efficacious than monotherapy alone for the treatment of diabetic peripheral neuropathy.

## 1. Introduction

Diabetic peripheral neuropathy occurs in over 50 percent of diabetic patients. It is associated with foot ulceration and is the leading cause of nontrauma foot amputations [1]. Approved treatments to slow progression or prevent diabetic peripheral neuropathy have failed in clinical trials. While intensive insulin therapy to maintain near-normal blood glucose can delay onset of symptoms in type 1 diabetes it is not effective in preventing the development of neuropathy or slowing the progression in type 2 diabetes [2, 3]. More efficacious compounds are necessary to address the growing burden of diabetic peripheral neuropathy.

Consumption of fish oil was shown to benefit those with cardiovascular diseases like congestive heart failure and has shown promise in type 2 diabetes, inflammatory diseases, stroke, depression, and anxiety disorders [4–8]. The ingestion of n-3 polyunsaturated fatty acids, eicosapentaenoic acid (EPA), and docosahexaenoic acid (DHA), those concentrated in fish oil, may be acted upon by lipoxygenases, acetylated cyclooxygenase, or cytochrome P450 enzymes producing serum elevations of various eicosanoid derivatives including resolvins of E and D series [5, 9]. Our current understanding of lipid derived health benefits implicates proresolving lipid mediators, such as resolvin D1, as the major curative entity produced from the metabolism of EPA and DHA [10]. In fact, results from many studies indicate that resolvins play a major endogenous role in the resolution of inflammation [11, 12]. There are very few side effects of fish oil consumption, individuals complain of fishy taste or breath, and no adverse events were reported in a large multicenter clinical trial [13]. Our group has shown that enrichment of diet with fish oil can improve outcome measures of type 1 and type 2 diabetes in Sprague-Dawley rats [14, 15]. Further, we have shown that treatment of C57Bl/6J mice with fish oil or 1 ng/g body weight of resolvin D1 ameliorates symptoms of peripheral neuropathy in a type 2 diabetic model [9].

Salsalate is a prodrug of salicylate and its primary mechanism of action is as a nonsteroidal anti-inflammatory that functions by inhibiting the synthesis of prostaglandins by way of inactivation of cyclooxygenase enzymes [16]. Experiments have established that salsalate, a nonacetylated salicylate, is insoluble in gastric juice and moves to the small intestine where it is hydrolyzed into two salicylic acid molecules. In clinical trials the main side effects of salsalate were tinnitus, headache, dizziness, and gastrointestinal discomfort, which occurred at higher doses and subside following withdrawal of treatment [17–20]. Salsalate produces fewer gastrointestinal bleedings when compared to acetylsalicylic acid due to less mucosal prostaglandin inhibition [21].

In this study we have used a streptozotocin induced type 1 diabetes mouse model, which we previously characterized [3], to assess the effectiveness of salsalate, menhaden oil, a fish oil enriched with n-3 fatty acids EPA and DHA, the combination of salsalate and menhaden oil, or the DHA derivative resolvin D1 in treating endpoints of diabetic peripheral neuropathy.

## 2. Materials and Methods

Chemicals used in this study came from Sigma-Aldrich chemical company (St. Louis, MO, USA), unless otherwise stated.

### 2.1. Animals

C57Bl/6J mice were purchased from Jackson Laboratories and housed in a certified animal care facility with food and water available ad libitum. Measures were taken to minimize the number of animals and their pain or discomfort. Experiments were conducted in accordance with international standards on animal welfare complying with institutional and National Institutes of Health animal use guidelines (ACURF protocol 1212258).

Twelve-week-old C57Bl/6J mice were divided into six groups, one vehicle treated control group and five groups treated with streptozotocin as previously described [22, 23]. Mice with a blood glucose level exceeding 300 mg/dL (16.7 mM) were considered diabetic (Aviva Accu-Chek, Roche, Mannheim, Germany). Following 8 weeks of untreated diabetes animals received 12 weeks of treatment by way of diet supplementation or a daily injection of resolvin D1. The targeted n for each group was 18 with an expectation of having 12 mice per group at the end of the study. Our overall success rate of induction of diabetes was 81%. The mice not having blood glucose greater than 300 mg/dL prior to initiation of treatment were excluded from the study. Several mice were also lost during the treatment phase. The caloric composition of the standard diet (Harlan Teklad, #7001, Madison, WI) or diet enriched in menhaden oil (Research Diets, New Brunswick, NJ) is provided in Table 1. The fatty acid composition of the standard and menhaden oil enriched diets is presented in Table 2 and was found to be similar to what we previously reported [14]. Control and one group of diabetic mice, the untreated diabetic group, received standard diet throughout the experiment. Three groups of diabetic mice received diet containing either menhaden oil (25% kcal fat, in diet), salsalate (250 mg/kg, in diet), or the combination of both menhaden oil and salsalate. Salsalate was mixed into the meal form of the standard or menhaden oil enriched diets and pelleted. The final group of diabetic mice was fed a standard diet and received a daily injection of resolvin D1 (1 ng/g body weight, i.p.) [9].

### 2.2. Behavioral Assays

Thermal nociceptive response was assessed in mice one week prior to euthanasia using the Hargreaves method with an IITC Life Science Inc. device (Woodland Hills, CA, model 390G) as previously described [3, 23, 24]. Five recordings were made with a 5-minute delay between recordings, the initial recording was discarded, and the remaining four were averaged and presented in seconds and served as the thermal nociceptive response latency.

### 2.3. Motor and Sensory Nerve Conduction Velocity

Mice were anesthetized with Nembutal (75 mg/kg, i.p., Abbott Laboratories, North Chicago, IL) and motor and sensory nerve conduction velocities were assessed as in previous experiments [3, 23, 24]. Motor nerve conduction velocity was calculated by using the stimulus artifact of the evoked potential, subtracting the latency measurement (in milliseconds) from the sciatic notch from the latency measurement of the Achilles tendon and dividing the difference by the distance between the two stimulating electrodes (measured in millimeters). Sensory nerve conduction velocity equaled the distance between stimulating and recording electrodes over the latency to initial peak negative deflection. Both motor and sensory nerve conduction velocity was reported in meters per second.

### 2.4. Corneal Innervation

The Rostock cornea module for the Heidelberg Retina Tomograph (Heidelberg Engineering, Vista, CA) was used for in vivo assessment of subepithelial nerves in the mouse cornea as described previously [3, 23]. Anesthetized mice were fitted to a stereotaxic mouse head holder (model 921-E, David Kopf Instruments, Tujunga, CA) and secured to a platform that allows for three-dimensional adjustments. GenTeal eye lubricant gel (Alcon, Fort Worth, TX) was applied to the lens and advanced forward to make contact with the mouse cornea epithelium. At least three nonoverlapping images of the subepithelial nerves were acquired per mouse and assessed for nerve length. Corneal nerve fiber length has proven to be the best morphological parameter in diagnosing diabetic neuropathy showing the lowest coefficient of variation [25, 26]. Corneal nerve fiber length is represented as a mean value of the nerve lengths measured from the images and expressed in mm/mm^2^.

During dissection the animal's cornea was isolated for immunofluorescent analysis as previously described [3, 23]. The cornea was dissected around the scleral-limbal region and fixed for 30 minutes in Zamboni's fixative. Corneas were blocked in phosphate-buffered saline (PBS) containing 0.2% Triton X-100, 2% goat serum, and 1% bovine serum albumin for 2 hours followed by overnight incubation with neuronal class *β*-III tubulin mouse monoclonal antibody at 1 : 1000 in 4°C (Covance, Dedham, MA). Following three 15-minute washes with PBS containing 0.2% Triton X-100, goat anti-mouse IgG_2a_ Alexa 488 secondary antibody was applied for 2 hours at room temperature (Invitrogen, Eugene, OR). Three more 15-minute washes in PBS containing 0.2% Triton X-100 preceded the mounting of the cornea (epithelium facing up) to a microscope slide in ProLong Gold (Life Technologies, Carlsbad, CA). A cover slip was placed on the cornea and sealed with clear fingernail polish.

Using Zeiss LSM710 Confocal Microscope running ZEN software (Carl Zeiss, Oberkochen, Germany) multiple images were collected to assess different nerve parameters in an attempt to more fully characterize the diseased cornea. Firstly, a 20x objective (Plan-Apochromat 20x/0.8) was used to make 8 × 8 tile scan z-stack (3400 *μ*m × 3400 *μ*m × 30 *μ*m; 4096 pixels × 4096 pixels × 30 pixels) images of the entire mouse cornea that were further processed to make a maximum projection intensity image for analysis. These images were analyzed for the amount of surface area covered by nerves using Imaris version 7.6.4 X64 software (Bitplane, Zurich, Switzerland). The surface option was used (parameters: smoothing enabled, surface grain size being equal to 0.833 *μ*m per pixel, no background elimination, the diameter of the largest sphere being 6.23 *μ*m, and thresholding being automatic) to determine the total surface area covered by nerves and is represented as a percentage of the total cornea surface area determined by manually tracing the cornea with a closed poly-line, as in previous experiments [9, 23]. Secondly, using a 63x objective (Plan-Apochromat 63x/1.4) a 3 × 3 tile scan z-stack (405 *μ*m × 405 *μ*m × 30.11 *μ*m; 1536 pixels × 1536 pixels × 78 pixels) was taken with optimum axial resolution to image the epithelial nerves of the cornea. These images were cropped to include only the epithelial nerves and subjected to volume analysis using Imaris version 7.6.4 X64 software (parameters: smooth was enabled, surface grain size equaled 0.187 *μ*m, no background elimination was used, diameter of the largest sphere was 0.701 *μ*m, and thresholding was automatic); the nerve volume is represented as a percentage of total volume as used for previous experiments [3, 9, 23]. For presentation purposes images were adjusted using Imaris and scale bars inserted with Fiji [27].

### 2.5. Skin Intraepidermal Nerve Fiber Density

Density of intraepidermal nerve fibers was determined as in previous experiments [3, 23]. Nerve profiles were imaged using a Zeiss LSM710 confocal microscope with a 40x objective (EC Plan-Neofluar 40x/0.75), counted by two independent investigators blinded to the sample condition, and profiles were normalized to the length of the epidermis in millimeters.

### 2.6. Blood and Serum Determinations

Mouse blood was collected from the right ventricle and serum was scrutinized for triglycerides, free fatty acids, cholesterol, and resolvin D1 using commercial kits as reported in previous experiments [3, 9, 23].

### 2.7. Data Analysis

Results are presented as mean ± standard error of the mean (SEM). One-way analysis of variance (ANOVA) and Sidak's multiple comparisons test were performed to determine group differences (GraphPad Prism 6, GraphPad, San Diego, CA). *p* < 0.05 was considered significant.

## 3. Results

Animals were diabetic for a total of 20 weeks and for the final 12 weeks selected animals received treatment via dietary supplementation of menhaden oil, salsalate, or menhaden oil plus salsalate or daily injection of 1 ng/g resolvin D1. As seen in Table 3, all diabetic mice failed to gain significant weight compared to control animals regardless of treatment and with only a slight nonsignificant addition of weight in the groups receiving menhaden oil supplementation (*F*(5,81) = 14.54, *p* < 0.0001). Throughout the experiment, diabetic animals had elevated blood glucose levels that were greater than in control mice and were not significantly impacted by treatment (*F*(5, 81) = 60.49, *p* < 0.0001 and *F*(5, 81) = 12.14, *p* < 0.0001, resp.).

Serum triglycerides and free fatty acids were not significantly changed in any of the groups.

Serum cholesterol levels were 20.0 ± 1.2 mg/dL in control mice but trended towards an increase in all diabetic groups (*F*(5,72) = 3.53, *p* < 0.01; diabetic group  26.8 ± 1.4, diabetic plus salsalate group 28.6 ± 4.0, and resolvin D1 group 27.3 ± 1.1 mg/dL). For mice receiving menhaden oil and menhaden oil plus salsalate serum cholesterol was increased over the control group (34.2 ± 3.8 and 32.0 ± 3.6 mg/dL, resp., versus 20.0 ± 1.2).

**Figure 1:**
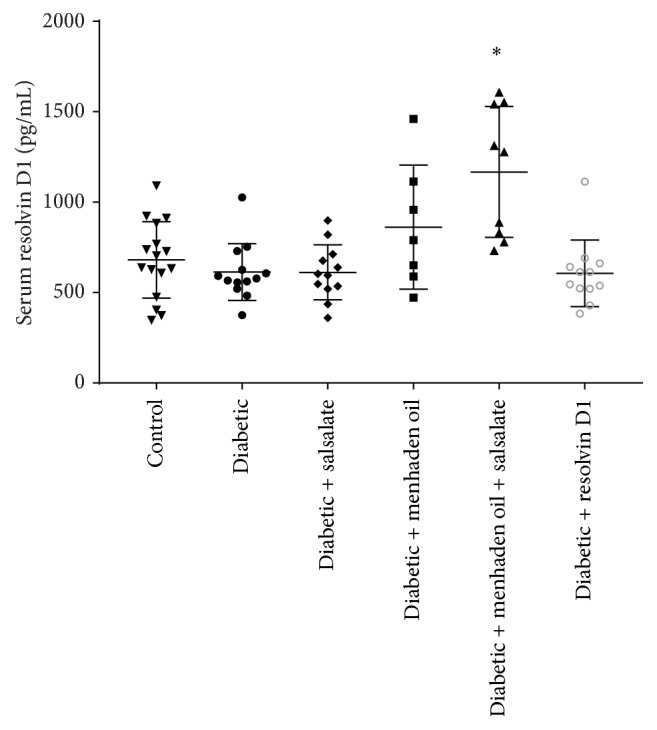
Scatter plot of serum resolvin D1 levels. Effects of 20 weeks of diabetes and 12 weeks of dietary treatment with salsalate, menhaden oil, menhaden oil plus salsalate, or resolvin D1 daily injections on serum levels of resolvin D1 in type 1 diabetic mice are presented. Serum levels are presented as mean ± SD and are in pg/mL serum. Each symbol per group represents a different animal. ^*∗*^ *p* < 0.05 to control group.

As seen in Figure 1, serum resolvin D1 levels trended to be elevated in diabetic mice receiving menhaden oil and elevated to a significant level in the menhaden oil plus salsalate group compared to control (*F*(5,60) = 8.13, *p* < 0.0001; menhaden oil 862 ± 130 pg/mL, menhaden oil plus salsalate 1151 ± 117 pg/mL, and control 680 ± 52 pg/mL) while the untreated diabetic group (diabetic group 613 ± 47 pg/mL) remained unchanged from control, salsalate, and resolvin D1 groups (714 ± 127, 612 ± 44 and 620 ± 63 pg/mL, resp.).

**Figure 2:**
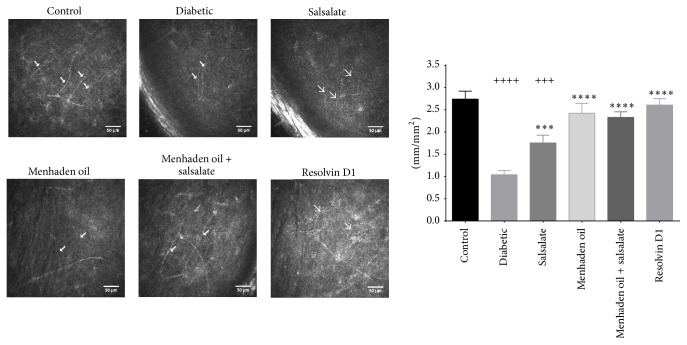
In vivo confocal microscopy was used to assess the effectiveness of drug treatment in diabetic mice. Subepithelial corneal innervation was scrutinized and images were analyzed for total nerve length. Arrows in the representative images indicate the location of subepithelial nerves. Data for innervation are presented as the mean ± SEM and shown in mm/mm^2^. The number of animals per group was the same as described in Table 3. ^+++^ *p* < 0.001 compared to control group, ^++++^ *p* < 0.0001 compared to control group, ^*∗∗∗*^ *p* < 0.001 compared to untreated diabetic group, and ^*∗∗∗∗*^ *p* < 0.0001 compared to untreated diabetic group.

**Figure 3:**
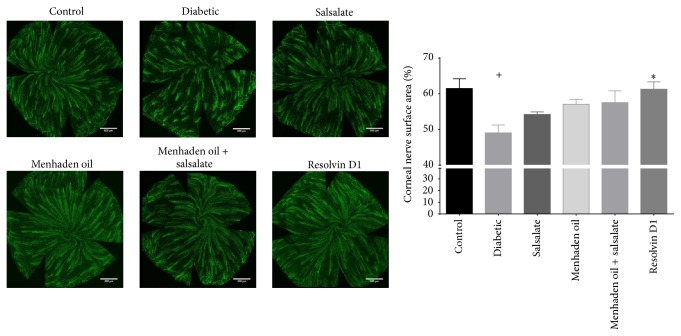
Immunohistochemical analysis of subepithelial corneal nerves using neuronal class *β*-III tubulin. A maximum intensity projection image was acquired as described in the Materials and Methods. Corneal nerve surface area was determined using Imaris software and the effects of treatment can be seen in the graph. Data are presented as mean percentage of nerve coverage ± SEM relative to the area of the entire cornea. The number of animals per group was the same as described in Table 3. ^+^ *p* < 0.05 compared to control group. ^*∗*^ *p* < 0.05 compared to untreated diabetic group.

The in vivo analysis of corneal nerves with corneal confocal microscopy is presented in Figure 2 [28]. Untreated diabetic mice presented with very little observable nerves in the subepithelial layer while control animals typically have several nerve fibers easily identified and measured (*F*(5,76) = 16, *p* < 0.0001). Following treatment with menhaden oil, menhaden oil plus salsalate, and resolvin D1 subepithelial corneal nerve occupancy appears similar to the control mice (2.4 ± 0.8, 2.3 ± 0.5, 2.6 ± 0.5, and 2.7 ± 0.8 mm/mm^2^, resp.) whereas salsalate treatment alone had marginal but significant improvement over diabetic animals (1.8 ± 0.6 and 1.0 ± 0.4 mm/mm^2^). Using an antibody to *β*-III tubulin we further examined the density of corneal nerves in the epithelial and subepithelial layers by immunohistochemistry.  Figure 3 shows that the surface area covered by corneal nerves in the subepithelial layer of diabetic mice is significantly reduced when compared to control (*F*(5,76) = 4.4, *p* = 0.0015; 49 ± 2.7 and 61 ± 2.2% area, resp.) and treatment with salsalate, menhaden oil, and menhaden oil plus salsalate provides a trend towards improvement while resolvin D1 treatment significantly increases nerve surface area over diabetic animals (54 ± 0.7, 57 ± 1.4, 58 ± 3.3, and 61 ± 2.1, resp.). As observed in the representative images untreated diabetic mice show reduced subepithelial nerve bundle length and compared to control mice a greater area of the cornea is devoid of *β*-III tubulin staining.

**Figure 4:**
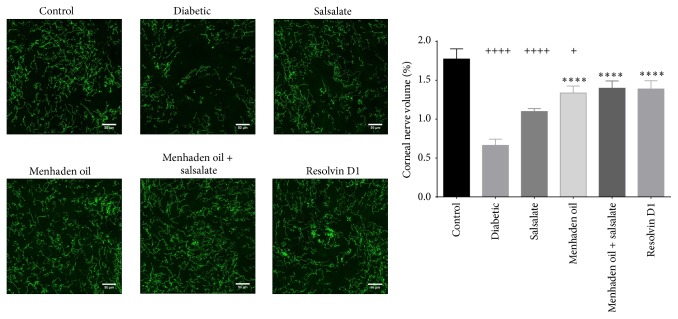
Immunohistochemical analysis of epithelial corneal nerves using neuronal class *β*-III tubulin. Confocal images were obtained as described in the Materials and Methods and assessed using Imaris software for the percentage of nerve volume relative to the total volume imaged. Data are presented in percentage of nerve volume relative to total volume imaged ± SEM. The number of animals per group was the same as described in Table 3. ^+^ *p* < 0.05 compared to control group, ^++++^ *p* < 0.0001 compared to control group, and ^*∗∗∗∗*^ *p* < 0.0001 compared to untreated diabetic group.

Figure 4 provides data and representative images of the density of corneal epithelial nerves over the central whorl region of the cornea. Control animals have a uniform epithelial innervation (1.8 ± 0.3% volume) and when compared to untreated diabetic animals we found the innervation volume significantly reduced (*F*(5,76) = 14, *p* < 0.0001). Treatment with menhaden oil, menhaden oil plus salsalate, or resolvin D1 provided significant benefits compared to untreated diabetic mice (1.3 ± 0.3, 1.4 ± 0.3, 1.4 ± 0.4, and 0.6 ± 0.3% volume, resp.) and salsalate alone showed a modest nonsignificant increase over untreated diabetic mice (1.1 ± 0.1% volume).

Following 20 weeks of hyperglycemia untreated diabetic mice had motor and sensory nerve conduction velocities significantly reduced when compared to control animals (MNCV *F*(5,81) = 12.47, *p* < 0.0001, and control 41.1 ± 1.5 versus diabetic group 27.7 ± 1.0 m/sec; SNCV *F*(5,81) = 21.49, *p* < 0.0001, and control 29.9 ± 0.7 versus diabetic group 22.5 ± 0.6) and treatment with menhaden oil, menhaden oil plus salsalate, and resolvin D1 produced significantly faster conduction velocities compared to untreated diabetic mice (MNCV 37.2 ± 1.1, 39.2 ± 1.0, and 38.8 ± 1.1, resp., versus 27.7 ± 1.0 m/sec; SNCV 29.5 ± 0.8, 30.2 ± 0.6, and 29.3 ± 0.5, resp., versus 22.5 ± 0.6) (Table 2). Treatment with salsalate alone significantly improved sensory nerve conduction velocities compared to untreated diabetic mice (27.0 ± 0.5 versus 22.5 ± 0.6 m/sec); however, motor nerve conduction velocity did not reach significance over untreated diabetic mice (35.2 ± 2.4 versus 27.7 ± 1.0 m/sec). Both motor and sensory nerve conduction velocity in diabetic mice treated with salsalate alone remained significantly reduced compared to control mice.

Untreated diabetic mice show a reduction in intraepidermal nerve profiles compared to control mice and treatment with salsalate, menhaden oil, menhaden oil plus salsalate, and resolvin D1 showed significantly more nerve profiles (*F*(5,81) = 44.20, *p* < 0.0001; 14.6 ± 0.4, 24.7 ± 0.7, 18.4 ± 0.4, 21.3 ± 0.5, 21.0 ± 0.6, and 20.4 ± 0.3 profiles/mm, resp.). All diabetic mice showed a reduced thermal sensitivity compared to control animals, except those treated with resolvin D1 (*F*(5,81) = 32.0, *p* < 0.0001; control 5.7 ± 0.2 versus diabetic group 8.6 ± 0.2 sec). Treatment with salsalate, menhaden oil, menhaden oil plus salsalate, and resolvin D1 significantly improved thermal sensitivity over untreated diabetic mice (7.3 ± 0.2, 6.6 ± 0.2, 6.7 ± 0.1, and 5.8 ± 0.2, resp., versus 8.6 ± 0.2 sec).

## 4. Discussion

Pharmaceutics used in the treatment of diabetic neuropathy range from nonsteroidal anti-inflammatory drugs such as aspirin, anticonvulsants like gabapentin and pregabalin, antidepressants such as tricyclic amitriptyline or serotonin-norepinephrine reuptake inhibitors like duloxetine and venlafaxine, and opioids like tramadol [29]. Many of the available treatments have shown promise for reducing pain yet with side effects such as sedation, fatigue, nausea, sweating, and peripheral edema, and respiratory depression patients are reluctant to comply with doctor recommendations. Complicating the matter, the typical diabetic patient uses more than one medicine which may hinder the identification of side effects and may lead to discontinuation of treatment. Most importantly, these drugs do not constitute a treatment for diabetic peripheral neuropathy and they merely target painful symptoms. In this experiment we have used a series of compounds that have a large therapeutic index and minimal side effects and targeted endpoints constituting a treatment to prevent or slow the progression of diabetic peripheral neuropathy.

Salsalate and its active metabolite salicylic acid are structurally and functionally similar to acetylsalicylic acid, but contrary to aspirin, salsalate passes through the stomach into the small intestine before being hydrolyzed into two salicylic acid molecules. Salicylic acid exerts its effects by reducing the generation of prostaglandins via cyclooxygenase inactivation [16]. In this study we extend previous findings by showing that salsalate provides benefit when used alone for the treatment of diabetes. As we show in Table 3, salsalate treated diabetic mice had improved motor and sensory nerve conduction velocities, salsalate benefited animals' thermal sensitivity, and the foot pad of salsalate treated animals had greater number of nerve profiles. These data indicate that salsalate alone reduced the pathological consequences of diabetic neuropathy.

In humans, salsalate has been used to treat the pain and inflammation associated with rheumatoid arthritis, osteoarthritis, and other rheumatic disorders [30, 31]. There are multiple noncyclooxygenase uses for salsalate in diabetes including results from randomized placebo-controlled studies showing salsalate reduced fasting blood glucose, HbA_1c_, and circulating triglycerides [18, 19, 32]. Furthermore, studies have shown salsalate treatment blocks free fatty acid mediated vascular insulin resistance [33] with improved glycemic control and with reduced inflammatory outcomes measures [17, 20]. Some beneficial metabolic effects may be mediated by increased energy expenditure from activated brown adipose tissue [34] and reduced microvascular complications via inhibition of inhibitor of kappa B kinase *β* (IKK*β*), the subsequent reduction in NF-*κ*B related genes, and increased nitric oxide (NO) preventing ischemic nerve damage [35, 36]. This study demonstrated for the first time that salsalate alone can improve diabetic neuropathic endpoints in a mouse model of type 1 diabetes even when treatment was delayed for eight weeks after the onset of hyperglycemia.

Fish oils, like menhaden oil, contain a high concentration of EPA and DHA and their consumption elevates the plasma concentration of proresolving inflammatory lipid mediators like resolvin D1 [9]. This study extends our previous findings by demonstrating the effectiveness of menhaden oil and resolvin D1 in alleviation of diabetic neuropathy outcome measures in a type 1 diabetes mouse model [9]. As seen in Table 4, diabetic mice receiving menhaden oil or resolvin D1 showed improved motor and sensory nerve conduction velocities and improved thermal sensitivity and more nerve profiles in the skin compared to untreated diabetic mice. Further, as shown in Figures 2, 3, and 4 diabetic mice receiving menhaden oil treatment or resolvin D1 presented with greater nerve coverage in the subepithelial layer, both by in vivo confocal microscopy and by immunohistochemistry, and had a greater volume of epithelial nerves in the corneal epithelium. We attribute these results in part to the increased circulating levels of proresolving lipid mediators found following dietary enrichment with fish oil or exogenous treatment with the derivate resolvin D1. It is important to recognize that we only measured circulating levels of resolvin D1. It is likely that other proresolving inflammatory lipid mediators such as resolvin E1 were also elevated following treatment with menhaden oil. Resolvin D1 levels were not found to be elevated in serum of diabetic mice treated exogenously with resolvin D1. This was likely due to the serum being collected at the termination of the study which was not performed until 24 h after the final resolvin D1 treatment. Resolvin D1 is rapidly converted into 17-oxoresolvin D1, which reduces its activity [37].

There are at least two ways to produce resolvin D1 from DHA. DHA may be acted upon by 15-lipoxygenase, peroxidases, 5-lipoxygenase, and hydrolases before the derivate resolvin D1 is generated. Additionally, aspirin-triggered resolvin D1 can be generated by acetylated cyclooxygenase followed by activity of a peroxidase and hydrolase [38]. We previously showed [9] that ingestion of menhaden oil will elevate serum levels of resolvin D1 and in this study we confirm this finding and extend the result by implicating salsalate as a possible agent for further enhancing the circulating concentration of proresolving lipid mediators, such as resolvin D1. This finding can be observed in Figure 1 where diabetic mice receiving a combination of menhaden oil and salsalate were found to have a resolvin D1 average of nearly three hundred pg/mL more than diabetic mice treated with menhaden oil alone. This indicates, for the first time, that salsalate, or its active molecule salicylic acid, triggers an elevation of resolvin D1 levels beyond that observed with menhaden oil treatment alone.

In this study, when animals received the combined treatment of salsalate and menhaden oil we did not observe an improvement in diabetic neuropathy outcome measures beyond monotherapy with menhaden oil. This was not surprising since the study was not designed to examine the potential for elevating lipid mediator levels following low dose fish oil combined with salsalate treatment. In this study we used the maximum concentration of menhaden oil possible for treatment of type 1 diabetic rodents [14]. And while the amount of fish oil tested in this study (25% kcal fat from fish oil) is not an unrealistic dose, we are pushing the limits of what a human will consume daily. Results from this study do provide rationale for a more comprehensive study to examine whether increased resolvin levels can be achieved with lower levels of dietary fish oil when combined with salsalate and if this combination can be as effective in treating diabetic neuropathy as the maximum dietary levels of fish oil.

## 5. Conclusions

The principle finding of our study is that anti-inflammatory compounds such as salsalate and dietary supplements such as fish oil can provide benefit to patients suffering from diabetic peripheral neuropathy. Whether the combination of salsalate and lower doses of fish oil may provide a similar benefit for treatment of diabetic neuropathy as the higher doses of fish oil will require further investigation.

## Figures and Tables

**Table 1:** Caloric composition of the diets.

Composition	Control Diet (Teklad 7001)	Menhaden oil Diet (Research Diets D11111603)
Protein (% total kcal)	34	20
Carbohydrate (% total kcal)	53	55
Fat (% total kcal)	13	25
Total (%)	100	100

kcal/g	3.0	4.2

**Table 2:** Fatty acid % composition of diets measured by gas chromatography.

Diet	16:0	16:1	18:0	18:1	18:2	20:5	22:6
Standard diet (3)	20 ± 2	2 ± 1	9 ± 2	29 ± 4	34 ± 4	<1	<1
Menhaden oil diet (3)	12 ± 2	5 ± 1	15 ± 2	15 ± 2	13 ± 2	15 ± 3	10 ± 1

Data are presented as the mean ± SEM. 16:0, palmitic acid; 16:1, palmitoleic acid; 18:0, stearic acid; 18:1, oleic acid; 18:2, linoleic acid; 20:5, eicosapentaenoic acid; and 22:6, docosahexaenoic acid. Numbers in parentheses indicate the number of experimental determinations.

**Table 3:** Effects of treatments of type 1 diabetes in C57Bl6/mice on change in body weight, blood glucose, and serum lipids.

Determination	Control (18)	Diabetic group (16)	Diabetic + salsalate group (12)	Diabetic + menhaden oil group (11)	Diabetic + salsalate + menhaden oil group (14)	Diabetic + resolvin D1 group (13)
Start weight (g)	25.5 ± 0.3	25.6 ± 0.3	25.6 ± 0.4	25.0 ± 0.5	25.0 ± 0.5	26.8 ± 0.4
Final weight (g)	30.6 ± 0.9	25.6 ± 0.3^a^	25.4 ± 0.5^a^	27.0 ± 0.5^a^	26.7 ± 0.3^a^	24.7 ± 0.3^a^
Blood glucose (mg/dL)	183.1 ± 5	491 ± 19^a^	463 ± 19^a^	546 ± 21^a^	491 ± 20^a^	480 ± 24^a^
Serum triglycerides (mg/dL)	9.4 ± 1.0	12.4 ± 0.9	10.6 ± 0.9	8.2 ± 0.6	9.8 ± 1.1	11.5 ± 0.9
Serum free fatty acids (mmol/L)	0.47 ± 0.08	0.41 ± 0.05	0.44 ± 0.06	0.26 ± 0.04	0.32 ± 0.06	0.38 ± 0.11
Serum cholesterol (mg/dL)	20.0 ± 1.2	26.8 ± 1.4	28.6 ± 4.0	34.2 ± 3.8^a^	32.0 ± 3.6^a^	27.3 ± 1.1

Data are presented as the mean ± SEM. ^a^ *p* < 0.05 compared to control mice. Numbers in parentheses indicate the number of experimental animals.

**Table 4:** Effect of treatments of type 1 diabetes in C57Bl/6J mice on motor and sensory nerve conduction velocity, intraepidermal nerve fiber density (IENFD), and thermal sensitivity.

Determination	Control (18)	Diabetic group (16)	Diabetic + salsalate group (12)	Diabetic + menhaden oil group (11)	Diabetic + salsalate + menhaden oil group (14)	Diabetic + resolvin D1 group (13)
Motor nerve conduction velocity (m/sec)	41.1 ± 1.5	27.7 ± 1.0^a^	35.2 ± 2.4^a^	37.2 ± 1.1^b^	39.2 ± 1.0^b^	38.8 ± 1.1^b^
Sensory nerve conduction velocity (m/sec)	29.9 ± 0.7	22.5 ± 0.6^a^	27.0 ± 0.5^ab^	29.5 ± 0.8^b^	30.2 ± 0.6^b^	29.3 ± 0.5^b^
IENFD (profiles/mm)	24.7 ± 0.7	14.6 ± 0.4^a^	18.4 ± 0.4^b^	21.3 ± 0.5^b^	21.0 ± 0.6^b^	20.4 ± 0.3^b^
Thermal sensitivity (sec)	5.7 ± 0.2	8.6 ± 0.2^a^	7.3 ± 0.2^ab^	6.6 ± 0.2^ab^	6.7 ± 0.1^ab^	5.8 ± 0.2^bc^

Data are presented as the mean ± SEM. ^a^ *p* < 0.05 compared to control mice. ^b^ *p* < 0.05 compared to diabetic mice. ^c^ *p* < 0.05 compared to diabetic + salsalate mice. Numbers in parentheses indicate the number of experimental animals.

## References

[1] Figueroa-Romero C., Sadidi M., Feldman E. L. (2008). Mechanisms of disease: the oxidative stress theory of diabetic neuropathy. *Reviews in Endocrine and Metabolic Disorders*.

[2] Fullerton B., Jeitler K., Seitz M., Horvath K., Berghold A., Siebenhofer A. (2014). Intensive glucose control versus conventional glucose control for type 1 diabetes mellitus. *The Cochrane Database of Systematic Reviews*.

[3] Yorek M. S., Obrosov A., Shevalye H. (2014). Effect of glycemic control on corneal nerves and peripheral neuropathy in streptozotocin-induced diabetic C57Bl/6J mice. *Journal of the Peripheral Nervous System*.

[4] De Caterina R., Madonna R., Bertolotto A., Schmidt E. B. (2007). n-3 fatty acids in the treatment of diabetic patients: biological rationale and clinical data. *Diabetes Care*.

[5] De Caterina R. (2011). n-3 fatty acids in cardiovascular disease. *The New England Journal of Medicine*.

[6] Calder P. C. (2015). Functional roles of fatty acids and their effects on human health. *Journal of Parenteral and Enteral Nutrition*.

[7] Zhang W., Wang H., Zhang H. (2015). Dietary supplementation with omega-3 polyunsaturated fatty acids robustly promotes neurovascular restorative dynamics and improves neurological functions after stroke. *Experimental Neurology*.

[8] Müller C. P., Reichel M., Mühle C., Rhein C., Gulbins E., Kornhuber J. (2015). Brain membrane lipids in major depression and anxiety disorders. *Biochimica et Biophysica Acta—Molecular and Cell Biology of Lipids*.

[9] Shevalye H., Yorek M. S., Coppey L. J. (2015). Effect of enriching the diet with menhaden oil or daily treatment with resolvin D1 on neuropathy in a mouse model of type 2 diabetes. *Journal of Neurophysiology*.

[10] Serhan C. N., Hong S., Gronert K. (2002). Resolvins: A family of bioactive products of omega-3 fatty acid transformation circuits initiated by aspirin treatment that counter proinflammation signals. *The Journal of Experimental Medicine*.

[11] Ji R.-R., Xu Z.-Z., Strichartz G., Serhan C. N. (2011). Emerging roles of resolvins in the resolution of inflammation and pain. *Trends in Neurosciences*.

[12] Kohli P., Levy B. D. (2009). Resolvins and protectins: mediating solutions to inflammation. *British Journal of Pharmacology*.

[13] Kromhout D., Giltay E. J., Geleijnse J. M. (2010). n-3 fatty acids and cardiovascular events after myocardial infarction. *The New England Journal of Medicine*.

[14] Coppey L. J., Davidson E. P., Obrosov A., Yorek M. A. (2015). Enriching the diet with menhaden oil improves peripheral neuropathy in streptozotocin-induced type 1 diabetic rats. *Journal of Neurophysiology*.

[15] Davidson E. P., Holmes A., Coppey L. J., Yorek M. A. (2015). Effect of combination therapy consisting of enalapril, *α*-lipoic acid, and menhaden oil on diabetic neuropathy in a high fat/low dose streptozotocin treated rat. *European Journal of Pharmacology*.

[16] Higgs G. A., Salmon J. A., Henderson B., Vane J. R. (1987). Pharmacokinetics of aspirin and salicylate in relation to inhibition of arachidonate cyclooxygenase and antiinflammatory activity. *Proceedings of the National Academy of Sciences of the United States of America*.

[17] Fleischman A., Shoelson S. E., Bernier R., Goldfine A. B. (2008). Salsalate improves glycemia and inflammatory parameters in obese young adults. *Diabetes Care*.

[18] Goldfine A. B., Fonseca V., Jablonski K. A. (2013). Salicylate (Salsalate) in patients with type 2 diabetes: a randomized trial. *Annals of Internal Medicine*.

[19] Goldfine A. B., Fonseca V., Jablonski K. A., Pyle L., Staten M. A., Shoelson S. E. (2010). The effects of salsalate on glycemic control in patients with type 2 diabetes: a randomized trial. *Annals of Internal Medicine*.

[20] Faghihimani E., Aminorroaya A., Rezvanian H., Adibi P., Ismail-Beigi F., Amini M. (2013). Salsalate improves glycemic control in patients with newly diagnosed type 2 diabetes. *Acta Diabetologica*.

[21] Cryer B., Goldschmidt M., Redfern J. S., Feldman M. (1990). Comparison of salsalate and aspirin on mucosal injury and gastroduodenal mucosal prostaglandins. *Gastroenterology*.

[22] Stavniichuk R., Shevalye H., Lupachyk S. (2014). Peroxynitrite and protein nitration in the pathogenesis of diabetic peripheral neuropathy. *Diabetes/Metabolism Research and Reviews*.

[23] Yorek M. S., Obrosov A., Shevalye H. (2015). Effect of diet-induced obesity or type 1 or type 2 diabetes on corneal nerves and peripheral neuropathy in C57Bl/6J mice. *Journal of the Peripheral Nervous System*.

[24] Coppey L., Davidson E., Lu B., Gerard C., Yorek M. (2011). Vasopeptidase inhibitor ilepatril (AVE7688) prevents obesity- and diabetes-induced neuropathy in C57Bl/6J mice. *Neuropharmacology*.

[25] Malik R. A., Kallinikos P., Abbott C. A. (2003). Corneal confocal microscopy: a non-invasive surrogate of nerve fibre damage and repair in diabetic patients. *Diabetologia*.

[26] Tavakoli M., Kallinikos P., Iqbal A. (2011). Corneal confocal microscopy detects improvement in corneal nerve morphology with an improvement in risk factors for diabetic neuropathy. *Diabetic Medicine*.

[27] Schindelin J., Arganda-Carreras I., Frise E. (2012). Fiji: an open-source platform for biological-image analysis. *Nature Methods*.

[28] Pritchard N., Edwards K., Russell A. W., Perkins B. A., Malik R. A., Efron N. (2015). Corneal confocal microscopy predicts 4-year incident peripheral neuropathy in type 1 diabetes. *Diabetes Care*.

[29] Javed S., Alam U., Malik R. A. (2015). Burning through the pain: treatments for diabetic neuropathy. *Diabetes, Obesity and Metabolism*.

[30] Montrone F., Caruso I., Cazzola M. (1989). Salsalate in the treatment of rheumatoid arthritis: a double-blind clinical and gastroscopic trial versus piroxicam. I. - Clinical trial. *The Journal of International Medical Research*.

[31] Bianchi Porro G., Petrillo M., Ardizzone S. (1989). Salsalate in the treatment of rheumatoid arthritis: a double-blind clinical and gastroscopic trial versus piroxicam II. *The Journal of International Medical Research*.

[32] Ariel D., Kim S. H., Liu A. (2015). Salsalate-induced changes in lipid, lipoprotein, and apoprotein concentrations in overweight or obese, insulin-resistant, nondiabetic individuals. *Journal of Clinical Lipidology*.

[33] Chai W., Liu J., Jahn L. A., Fowler D. E., Barrett E. J., Liu Z. (2011). Salsalate attenuates free fatty acid-induced microvascular and metabolic insulin resistance in humans. *Diabetes Care*.

[34] van Dam A. D., Nahon K. J., Kooijman S. (2015). Salsalate activates brown adipose tissue in mice. *Diabetes*.

[35] Krishnan M., Janardhanan P., Roman L. (2015). Enhancing eNOS activity with simultaneous inhibition of IKK*β* restores vascular function in Ins2Akita+/- type-1 diabetic mice. *Laboratory Investigation*.

[36] McCarty M. F. (2010). Salsalate may have broad utility in the prevention and treatment of vascular disorders and the metabolic syndrome. *Medical Hypotheses*.

[37] Orr S. K., Colas R. A., Dalli J., Chiang N., Serhan C. N. (2015). Proresolving actions of a new resolvin D1 analog mimetic qualifies as an immunoresolvent. *American Journal of Physiology—Lung Cellular and Molecular Physiology*.

[38] Serhan C. N., Petasis N. A. (2011). Resolvins and protectins in inflammation resolution. *Chemical Reviews*.

